# The Influence of Irrigation during the Finishing and Polishing of Composite Resin Restorations—A Systematic Review of In Vitro Studies

**DOI:** 10.3390/ma14071675

**Published:** 2021-03-29

**Authors:** João Paulo Silva, Ana Coelho, Anabela Paula, Inês Amaro, José Saraiva, Manuel Marques Ferreira, Carlos Miguel Marto, Eunice Carrilho

**Affiliations:** 1 Institute of Integrated Clinical Practice, Faculty of Medicine, University of Coimbra, 3000-075 Coimbra, Portugal; joao_silva2009@hotmail.com (J.P.S.); anabelabppaula@sapo.pt (A.P.); ines.amaros@hotmail.com (I.A.); ze-93@hotmail.com (J.S.); eunicecarrilho@gmail.com (E.C.); 2 Area of Environment Genetics and Oncobiology (CIMAGO), Coimbra Institute for Clinical and Biomedical Research (iCBR), Faculty of Medicine, University of Coimbra, 3000-548 Coimbra, Portugal; m.mferreira@netcabo.pt (M.M.F.); cmiguel.marto@uc.pt (C.M.M.); 3 Clinical Academic Center of Coimbra (CACC), 3004-561 Coimbra, Portugal; 4 Institute of Endodontics, Faculty of Medicine, University of Coimbra, 3000-075 Coimbra, Portugal; 5 Institute of Biophysics, Faculty of Medicine, University of Coimbra, 3004-548 Coimbra, Portugal; 6 Institute of Experimental Pathology, Faculty of Medicine, University of Coimbra, 3004-548 Coimbra, Portugal

**Keywords:** finishing and polishing, composite resins, irrigation, roughness, microhardness

## Abstract

The surface smoothness of composite restorations affects not only their esthetic appearance but also other properties. Thus, rough surfaces can lead to staining, plaque accumulation, gingival irritation, recurrent caries, abrasiveness, wear kinetics, and tactile perception by the patient. The aim of this study was to evaluate the influence of irrigation during the finishing and polishing of composite resin restorations. A systematic search of the PubMed, Cochrane Library, EMBASE, Web of Science, and Clinical Trials databases was conducted. Papers published up to 11 February 2021 were considered. The quality of each study was assessed using the modified Consolidated Standards of Reporting Trials checklist for reporting in vitro studies on dental materials. No clinical studies were identified. Six in vitro studies were included, reporting changes in physical and esthetic properties. After performing a methodological quality assessment of the studies, some limitations were identified, the main limitation being the heterogeneous methodology across studies. The evidence resulting from this systematic review did not favor either wet or dry finishing/polishing procedures. There is a clear need for well-designed studies focusing on the comparison of dry/wet finishing/polishing with standard protocols to evaluate the differences among different materials and methods.

## 1. Introduction

Composite resins have been increasingly used for direct restoration of both anterior and posterior teeth because of their optimal esthetics, improved physical and mechanical properties, availability of efficient bonding systems, and public concerns over amalgam use [1,2].

The esthetic appearance of the composite resin restorations is potentiated by finishing and polishing procedures. Finishing is related to the contouring, shaping, and smoothing of the restoration to give anatomical contours and remove excess material at the interface. After finishing, polishing is performed to achieve a surface with high luster and enamel-like texture [3].

The surface smoothness of the composite restorations affects not only their esthetic appearance but also other properties. Thus, rough surfaces can lead to staining, plaque accumulation, gingival irritation, recurrent caries, abrasiveness, wear kinetics, and tactile perception by the patient [4].

Finishing and polishing are frictional processes and thus produce heat. Excess heat may affect the interface between the tooth and adhesive bond [5], and also damage the pulp [6]. For this reason, the need for lubrication/coolant is a fundamental aspect.

Several studies have evaluated the effect of different finishing and polishing procedures on surface roughness, hardness, and temperature rise of composites [7,8,9]. However, there is no consensus in the literature about the difference between dry or wet finishing/polishing on the surface characteristics of composites.

Thus, the objective of this systematic review was to evaluate the influence of irrigation during the finishing and polishing of composite resin restorations, answering the following population, intervention, comparison, and outcome (PICO) question, “Does irrigation during the finishing and polishing steps influence the properties of composite resins?”, described in detail in Table 1.

## 2. Materials and Methods

This systematic review was performed following the Preferred Reporting Items for Systematic Reviews and Meta-Analysis protocol (PRISMA-P).

### 2.1. Search Strategy

The studies included in this systematic review were obtained from the MEDLINE (accessed through PubMed), Cochrane Library, EMBASE, Web of Science, and Clinical Trials databases. Gray literature was not included in this review. No restrictions on the year of publication, region, or language were considered. Articles published between January 1977 and 11 February 2021 were retrieved. The search terms used for each database are presented in Table 2.

The duplicate references were removed automatically using Mendeley (RELX Group, London, UK) and then checked manually by two authors.

The initial selection of the articles to be included was accomplished by two independent authors reading the titles and abstracts. Afterwards, the full text of each potentially relevant study was retrieved and also independently analyzed by two authors. The opinion of a third author was consulted when necessary.

The references of the included studies were searched to identify additional relevant references.

### 2.2. Inclusion and Exclusion Criteria

For this systematic review, only studies which met the following inclusion criteria were selected: (a) clinical studies or in vitro studies with resin blocks or extracted teeth; (b) evaluation on composite resins; (c) use of polishing systems with and/or without coolant; (d) studies that evaluated one or more of the following properties—color, roughness, microhardness, microleakage, gloss, temperature, and particle and substance release (e.g., bisphenol-A).

Exclusion criteria were as follows: (a) in vivo studies; (b) materials other than composite resins; (c) studies that did not evaluate the intended parameters; and (d) studies lacking the comparison of wet and dry finishing/polishing procedures using the same finishing/polishing system.

### 2.3. Data Extraction

The studies that fulfilled the inclusion criteria were processed for data extraction. The data were recorded as follows: first author and year of publication, groups and number of samples, materials, and time used per disc during polishing. The following outcomes were extracted: roughness (μm), microhardness, color change (ΔE), and temperature rise (°C).

The extraction of the information was done by two independent authors using a standard form. A consensus meeting was always held to confirm the agreement and to resolve any disagreement between the reviewers.

### 2.4. Quality Assessment

The evaluation of the methodological quality of the included studies is essential for understanding the results. Therefore, the quality of each in vitro study was assessed using the modified Consolidated Standards of Reporting Trials (CONSORT) checklist [10] for reporting in vitro studies on dental materials. When applying this checklist, items 5 to 9 could not be evaluated since these are designed to evaluate sample standardization (no author assessed this parameter). Two independent authors assessed the quality of the studies independently, and any disagreement was solved through discussion and consensus. The opinion of a third author was consulted when necessary.

## 3. Results

### 3.1. Study Selection

**Figure 1:**
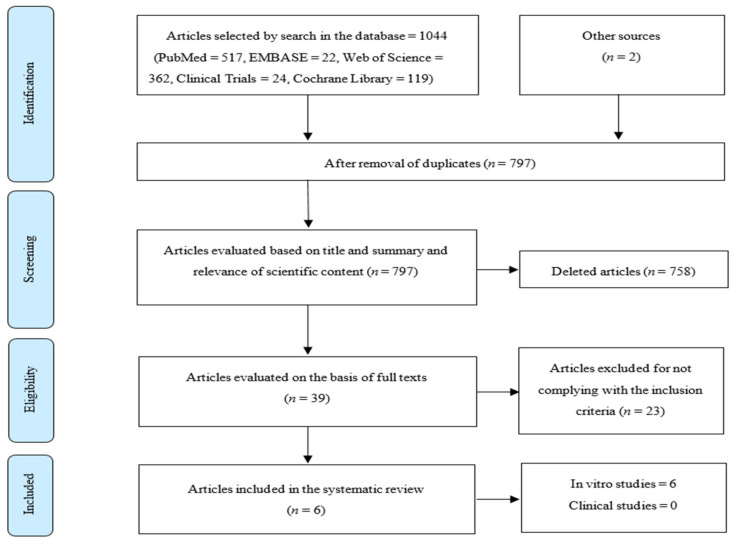
Preferred Reporting Items for Systematic Reviews and Meta-Analysis (PRISMA) flow diagram of the study selection process.

The PRISMA flow diagram of the study selection is shown in Figure 1.

A total of 1044 studies were identified through the search in the referred databases. After the removal of duplicates, a total of 797 articles were obtained, of which 39 were selected after reading the titles and abstracts. The full-text reading led to the exclusion of 23 articles when submitted to the inclusion and exclusion criteria. Two articles were included after analysis of the reference lists of the selected studies. No clinical studies were identified. Six in vitro studies were considered for analysis (Table 3).

### 3.2. Study Characteristics

Regarding the year of publication, the oldest study was published in 1991 [11], and the most recent one was published in 2019 [15].

All studies evaluated the finishing/polishing under both wet and dry conditions. Kaminedi et al. [13] also studied the differences between immediate and delayed finishing/polishing.

Regarding the finishing/polishing system, Sof-Lex™ discs (3M ESPE, Minneapolis, MN, USA) were used in five studies [11,12,13,14,15], and Super-Snap^®^ discs (Shofu Dental, San Marcos, CA, USA) were used by Jones et al. [9]. Kaminedi et al. [13] used diamond finishing burs (Diatech, Coltene, Geneve, Switzerland) in the finishing procedures and de Freitas et al. [15] employed a multilaminated carbide bur (48L-010: Angelus, Londrina, Brazil) and Sof-Lex™ spirals in the finishing/polishing procedures. The time spent using each disc varied widely among studies (10–30 s).

The included studies reported results on different types of materials including macrofilled [11], microfilled [11], hybrid [9,11,13], microhybrid [12,14], nanofilled [12], and nanohybrid [13,14,15] composites.

A summary of conclusions from the included studies can be found in Table 4.

### 3.3. Quality Assessment

Methodological quality assessment outcomes are presented in Table 5. All studies presented results for each experimental group; however, none of them referred to confidence intervals. Only one study [15] reported study limitations and sources of potential bias (item 12).

## 4. Discussion

High-quality finishing and polishing of dental restorations are essential aspects of clinical restorative procedures that enhance both esthetics and longevity of restored teeth [3]. Aiming to have the best finishing and polishing technique, it is vital to know not only if the irrigation improves the quality of the final result or not but also if it changes the material properties or damages the tooth (e.g., triggered by temperature rise).

The surface roughness of composite resins depends not only on intrinsic but also on extrinsic factors. Intrinsic factors include the type of material, type of filler, shape, size, and distribution of filler particles, degree of polymerization, resin matrix composition, and durability of filler/matrix bond [16]. Extrinsic factors are related to the finishing and polishing techniques and include the flexibility and geometrical shape of polishing tools, the hardness of abrasive particles, operator skills, and the way each technique is carried out [17]. Regarding the intrinsic factors and by following the general belief that composites with smaller filler particles prevent the wear of the resin matrix and minimize the surface alteration deriving from the particles’ detachment, it was expected that nanofilled and nanohybrid composites allowed for better surface smoothness. However, there is a low-level evidence attesting it [18]. As an extrinsic factor, different finishing and polishing tools (Sof-Lex™ discs [11,12,13,14,15], Super Snap^®^ discs [9], spirals [15], and burs [13,15]) and their manipulation (including the time per disc) may have contributed to the different results across studies.

When finishing and polishing procedures are performed without lubrication, a rise in materials temperature due to frictional forces is expected. When a sample is dry finished, a temperature rise at the surface level may cause localized softening and melting (exceeded glass transition point). This may lead to smearing of the resin over any exposed particles, making the particle-like appearance not so noticeable, and the surface smoother [9,11,12], which supports the results of Dodge et al. [11], Cardoso et al. [12], and Jones et al. [9]. However, dry finishing and polishing were revealed to be less successful (higher surface roughness) in other studies [13,14,15] and this can be supported by the fact that the abrasive particles separated from the polishing tool may be embedded into the composite’s surface. Furthermore, accumulation of separated particles on the surface of polishing tools can decrease its efficiency when attempting to smooth the surface [11]. On the other hand, the heat generated during dry finishing and polishing is high and can degrade the filler/matrix bond and result in separation of filler particles from the matrix and subsequently increase the surface roughness [5]. Moreover, other factors could explain these variable results, such as the application time and the variability among different operators regarding the applied load and speed of finishing and polishing [19]. The grit size of the polishing discs may also contribute to the changes in surface temperature, since when the disc is switched from one with a greater grit size to another one with a smaller grit, the temperature does not decrease, in fact, there is a temperature rise [9]. However, according to Lloyd et al. [20], this temperature rise is not hazardous for dental pulp because composites are heat insulators, and the generated heat during dry finishing and polishing is confined to the composite surface such that at 0.2 mm depth from the composite surface, the temperature does not exceed 10 °C.

Surface hardness is another important material property which correlates well with compressive strength, resistance to intra-oral softening, and conversion degree [21,22]. Low surface hardness values are largely related to inadequate wear resistance and can lead to failure of the restoration [23]. Composite hardness depends on several factors such as type and shape of filler particles, their composition and distribution, percentage of filler particles, and resin type. Reduction in the hardness of filler particles directly decreases the hardness of composite [24]. Kaminedi et al. [13] reported that although the finishing under coolant resulted in the highest surface hardness for micro hybrid resin (Filtek™ Z250), dry finishing groups showed a significant increase in surface hardness of the nano-composite resin material and a non-significant increase of hardness of the surfaces of hybrid composite materials. Furthermore, Nasoohi et al. [14] reported that hardness of all composite samples also increased when performing dry finishing. These results can be explained by the fact that hardness of composite increases by raising the temperature up to 60 °C, which is due to increased cross-linking between polymer chains [25]. Moreover, infrared tomography assessments have shown that the temperature at the surface of composites submitted to dry finishing and polishing is 140 °C or higher [14]. That temperature rise increases cross-linking and hardness because the temperature is higher than the glass-transition temperature of resin content [26].

Color change over time represents a problem for composite resin restorations. It depends on several factors, such as oral beverage and food colorant ingestion, poor hygiene, type of composite resin, the restorative technique used, as well as the finishing and polishing technique (surface roughness) [15,27,28]. Despite having only studied the Filtek™ Z-350 XT, de Freitas et al. [15] observed variations depending on the type of finishing and polishing technique, showing the importance of using irrigation to reduce color change, mainly when spirals are used.

Even though in vitro research cannot accurately reproduce the oral environment, only these experiments are able to provide information on the tribological properties of composite surfaces, which is why only in vitro studies were considered for inclusion in this review. However, after performing a methodological quality assessment of the included studies, some limitations were identified, the main limitation being the heterogeneous methodology across studies. Not all of them evaluated the same type of composite resin, there were different finishing/polishing materials and methods, and only four [11,12,13,14] and two [11,15] of them studied microhardness and color change, respectively. Furthermore, the majority of the studies evaluated a small number of samples in each group (n)—Jones et al. [9] had several experimental groups with a sample size of just one sample per group. Thus, this methodological variability observed among the included studies prevents us from drawing conclusions, making it vital to conduct further experiments with clear and established protocols in order to allow future comparison among a wider range of studies. As such, there is a clear need for researchers to conduct well-designed studies focusing on the comparison of dry vs. wet finishing/polishing with standard protocols to evaluate the differences among different materials and methods.

## 5. Conclusions

Different finishing and polishing protocols influence microhardness, roughness, color, and surface temperature of resin composites. However, the evidence resulting from this systematic review did not favor either wet or dry finishing/polishing procedures.

## Figures and Tables

**Table 1:** Population, intervention, comparison, and outcome (PICO) question.

Parameter	Description
P	Composite resin restorations
I	Wet finishing/polishing
C	Dry finishing/polishing
O	Changes in physical and esthetic properties

P-Population; I-Intervention; C-Comparison; O-Outcome.

**Table 2:** Search terms used in each one of the databases.

Database	Search Terms
PubMed	((((((irrigation[Title/Abstract]) OR (water[Title/Abstract])) OR (coolant[Title/Abstract])) OR (wet[Title/Abstract])) OR (dry[Title/Abstract])) AND ((((composite resins[MeSH Terms]) OR (resin*[Title/Abstract])) OR (composite restoration*[Title/Abstract])) OR (composite[Title/Abstract]))) AND (((((dental polishing[MeSH Terms]) OR (polishing[Title/Abstract])) OR (polish[Title/Abstract])) OR (finishing[Title/Abstract])) OR (finish[Title/Abstract]))
Cochrane Library	#1 (irrigation):ti,ab,kw #2 (water):ti,ab,kw #3 (coolant):ti,ab,kw #4 (wet):ti,ab,kw #5 (dry):ti,ab,kw #6 #1 OR #2 OR #3 OR #4 OR #5 #7 MeSH descriptor: [Dental Polishing] explode all trees #8 (polishing):ti,ab,kw #9 (polish):ti,ab,kw #10 (finishing):ti,ab,kw #11 (finish):ti,ab,kw #12 #7 OR #8 OR #9 OR #10 OR #11 #13 MeSH descriptor: [Composite Resins] explode all trees #14 (resin*):ti,ab,kw #15 (composite restoration*):ti,ab,kw #16 (composite):ti,ab,kw #17 #13 OR #14 OR #15 OR #16 #18 #6 AND #12 AND #17
Embase	(coolant:ab,ti OR wet:ab,ti OR water:ab,ti OR irrigation:ab,ti OR dry:ab,ti OR ‘irrigation’/exp OR ‘irrigation solution’/exp OR ‘water’/exp) AND (resin:ab,ti OR resins:ab,ti OR ‘composite restoration’:ab,ti OR composite: ab,ti OR ‘resin’/exp OR ‘composite material’/exp) AND (‘dental polishing’: ab,ti OR polishing:ab,ti OR finishing: ab,ti OR polish:ab,ti OR finish:ab,ti OR ‘polishing’/exp)
Web of Science	TS = (irrigation OR water OR coolant OR wet OR dry) AND TS = (polishing OR polish OR finishing OR finish) AND TS = (resin* OR composite restoration* OR composite) AND TS = (dentistry OR dental)
Clinical Trials	(irrigation OR water OR coolant OR wet OR dry) AND (polishing OR polish OR finishing OR finish) AND (resin* OR composite restoration* OR composite) AND (dentistry OR dental)

**Table 3:** Summary of the included studies.

Authors, Year	Groups (n)	Methodology		Results				Results with Statistical Significance (*p* < 0.05)
		Materials	Time per Disc	Roughness (μm)	Microhardness	Color Hange (∆E)	Temperature Rise (°C)	
Dodge et al., 1991 [11]	Wet G_1_: Prisma-fil^®^ (Dentsply Sirona, York, PA, USA) (6); G_2_: Silux™ (3M, Minneapolis, MN, USA) (6); G_3_: Herculite™ (Kerr/Sybron, Orange, CA, USA) (6); G_4_: Visio-Dispers (ESPE/Premier, Schaumburg, IL, USA) (6) Dry G_5_: Prisma-fil^®^ (6); G^6^: Silux™ (6); G_7_: Herculite™ (6); G_8_: Visio-Dispers (6)	Sof-Lex™ finishing disks: Coarse, medium, fine, superfine (3M, USA)	30 s	G_1_: 1.98; G_2_: 3.15; G_3_: 2.13; G_4_: 5.39; G_5_: 2.49; G_6_: 2.49; G_7_: 2.13; G_8_: 2.44	G_1_: 66.1; G_2_: 23.7; G_3_: 64.3; G_4_: 39.0; G_5_: 48.7; G_6_: 27.3; G_7_. 63.1; G_8_: 41.4 (KHN)	**After 1 week:** G_1_: 1.56; G_2_: 1.64; G_3_: 2.01; G_4_: 1.82; G_5_: 0.94; G_6_: 2.15; G_7_: 2.14; G_8_: 0.59 **After 5 weeks:** G_1_: 1.30; G_2_: 2.61; G_3_: 1.92; G_4_: 0.49; G_5_: 0.78; G_6_: 2.65; G_7_: 2.52; G_8_: 1.73		**Roughness:** G_4_ > G_8_ **Color change** **(0–5 weeks):** G_1_ G_3_ G_6_
Cardoso et al., 2005 [12]	Wet G_1_: Filtek™ Supreme (3M, Minneapolis, MN, USA) (20); G_2_: Filtek™ Z250 (3M, Minneapolis, MN, USA) (20) Dry G_3_: Filtek™ Supreme (20); G_4_: Filtek™ Z250 (20)	Sof-Lex™: coarse, medium, fine, ultrafine	30 s	G_1_: 0.6 ± 0.2 G_2_: 0.2 ± 0.1 G_3_: 0.2 ± 0,1 G_4_: 0.1 ± 0.0	G_1_: 76.0 ± 9.3 G_2_: 75.3 ± 20.2 G_3_: 71.5 ± 15.4 G_4_: 74.9 ± 16.4 (KHN)			**Roughness:** G_1_ > G_3_ G_2_ > G_4_
Jones et al., 2007 [9]	Filtek™ Z100 (3M, USA) Wet G_1_: coarse (1); G_2_: medium (1); G_3_: fine (1); G_4_: super-fine (1) Dry G_5_: coarse (1); G_6_: medium (1); G_7_: fine (1); G_8_: super-fine (1)	Super-Snap^®^ discs: coarse, medium, fine, superfine (Shofu Dental, USA)	22 s	G_1_: 0.37; G_2_: 0.34; G_3_: 0.26; G_4_: 0.20; G_5_: 0.23; G_6_: 0.12; G_7_: 0.09; G_8_: 0.06			G_1_: 0 G_2_: 0 G_3_: 0 G_4_: 0 G_5_: 11 G_6_: 16 G_7_: 13 G8: 14	**Roughness:** G_1–4_ > G_5–8_ **Temperature rise:** G_5–8_ > G_1–4_
Kaminedi et al., 2014 [13]	No finishing/polishing G_1_:Filtek™ Z250 (10); G_2_:Filtek™ Z350 (3M, Minneapolis, MN, USA) (10) Immediate wet F + dry P G_3_: Filtek™ Z250 (10); G_4_:Filtek™ Z350 (10) Wet F + dry P, after 15 min G_5_: Filtek™ Z250 (10); G_6_:Filtek™ Z350 (10) Wet F + dry P, after 24h G_7_: Filtek™ Z250 (10); G_8_:Filtek™ Z350 (10) Dry F + dry P G_9_: Filtek™ Z250 (10); G_10_: Filtek™ Z350 (10)	F- diamond finishing burs (Diatech, Genève, Switzerland) P- Sof-Lex™: medium, fine, superfine	F- 10 s P- 20 s	G_1_: 153.6 ± 17.7 G_2_: 126.7 ± 3.6 G_3_: 157.0 ± 8.5 G_4_: 142.9 ± 14.5 G_5_: 177.2 ± 20.2 G_6_: 148.5 ± 8.6 G_7_: 188.8 ± 18.4 G_8_: 156.6 ± 6.7 G_9_: 181.2 ± 15.4 G_10_: 183.9 ± 21.3	G_1_: 65.4 ± 7.8 G_2_: 67.7 ± 4.5 G_3_: 77.6 ± 7.5 G_4_: 67.1 ± 3.5 G_5_: 77.6 ± 7.5 G_6_: 61.3 ± 3.2 G_7_: 77.6 ± 7.5 G_8_: 65.9 ± 5.6 G_9_: 65.4 ± 7.8 G_10_: 69.0 ± 7.1 (HV)			**Roughness:** G_7_ > G_9_ > G_5_ > G_3_ > G_1_ G_10_ > G_8_ > G_6_ > G_4_ > G_2_ **Microhardness: ** G_10_ > G_2_ > G_4_ > G_8_ > G_6_ G_3_ > G_9_ > G_5_ > G_1_ > G_7_
Nasoohi et al., 2017 [14]	No F/P G_1_: Aelite™ Aesthetic Enamel (BISCO, Schaumburg, IL, USA) (10); G_2_: Aelite™ All Purpose Body (BISCO, Schaumburg, IL, USA) (10); G_3_: Grandio (Voco, Cuxhaven Germany) (10); G_4_: Polofil Supra (Voco, Cuxhaven Germany) (10) Wet F&P G_5_: Aelite™ Aesthetic Enamel (10); G_6_: Aelite™ All Purpose Body (10); G_7_: Grandio (10); G_8_: Polofil Supra (10) Dry F&P G_9_: Aelite™ Aesthetic Enamel (10); G_10_: Aelite™ All Purpose Body (10); G_11_: Grandio (10); G_12_: Polofil Supra (10)	Sof-Lex™ Pop-On Discs: coarse, medium, fine, ultrafine	20 s	G_1_: 0.02 ± 0.01 G_2_: 0.11 ± 0.01 G_3_: 0.15 ± 0.01 G_4_: 0.04 ± 0.01 G_5_: 0.13 ± 0.02 G_6_: 0.16 ± 0.01 G_7_: 0.05 ± 0.01 G_8_: 0.32 ± 0.02 G_9_: 0.43 ± 0.02 G_10_: 0.03 ± 0.01 G_11_: 0.13 ± 0.01 G_12_: 0.17 ± 0.01	G_1_: 61.00 ± 2.06 G_2_: 75.68 ± 2.09 G_3_: 94.37 ± 2.99 G_4_: 63.97 ± 2.54 G_5_: 78.20 ± 2.23 G_6_: 96.78 ± 2.10 G_7_: 115.09 ± 6.56 G_8_: 163.75 ± 2.86 G_9_: 199.92 ± 4.47 G_10_: 61.23 ± 1.92 G_11_: 76.15 ± 2.33 G_12_: 96.20 ± 3.75 (HV)			**Roughness:** G_9–12_ > G_5–8_ **Microhardness:** G_9–12_ > G_5–8_
de Freitas et al., 2019 [15]	Filtek™ Z-350 XT (3M ESPE, Minneapolis, MN, USA) G_1_: polyester strip (10); G_2_: no P (10) Wet G_3_: abrasive disks (10); G_4_: spirals (10); G_5_: abrasive disks + spirals (10); G_6_: abrasive disks + multilaminated carbide bur + spirals (10) Dry G_7_: abrasive disks (10); G_8_: spirals (10); G_9_: abrasive disks + spirals (10); G_10_: abrasive disks + multilaminated carbide bur + spirals (10)	Abrasive disks Sof-Lex™: coarse, medium, fine, ultrafine Spirals Sof-Lex™: pre-polishing, diamond polishing (3M ESPE, Minneapolis, MN, USA) Multilaminated carbide bur 48L-010 (Angelus, Londrina, Brazil)	N/A (20 x)	G_1_: 0.24 ± 0.14 G_2_: 0.8 ± 0.15 G_3_: 0.17 ± 0.05 G_4_: 0.06 ± 0.03 G_5_: 0.08 ± 0.03 G_6_: 0.04 ± 0.02 G_7_: 0.22 ± 0.14 G_8_: 0.51 ± 0.13 G_9_: 0.07 ± 0.04 G_10_: 0.10 ± 0.02		**0–7 days:** G_1_: 1.6 ± 0.7 G_2_: 3.9 ± 1.6 G_3_: 2.4 ± 1.1 G_4_: 1.9 ± 0.3 G_5_: 2.0 ± 0.3 G_6_: 1.8 ± 0.3 G_7_: 1.5 ± 0.6 G_8_: 2.2 ± 0.4 G_9_: 2.4 ±0.9 G_10_: 1.9 ±0.4 **0–14 days:** G_1_: 2.3 ± 1.1 G_2_: 3.5 ± 1.3 G_3_: 2.8 ± 1.1 G_4_: 2.0 ± 0.1 G_5_: 2.5 ± 0.5 G_6_: 2.8 ± 0.1 G_7_: 2.4 ± 0.9 G_8_: 2.9 ± 0.8 G_9_: 3.4 ± 0.5 G_10_: 2.8 ± 0.7		**Roughness:** G_8_ > G_4_; G_10_ > G_6_ **Color Change:** 0–14 days: G_8_ > G_4_; G_9_ > G_5_

F–finishing; P–polishing; s–seconds; KHN–Knoop hardness number; HV–Vickers microhardness.

**Table 4:** Summary of conclusions from the included studies.

Authors, Year	Conclusion of the Study	Results Favor Finishing/Polishing	
		Dry	Wet
Dodge et al., 1991 [11]	“Dry finishing of composites was superior or equal to wet finishing in all tests except for the color change in Silux”	x	
Cardoso et al., 2005 [12]	The composite resin presented better polishing results when it was executed without coolant	x	
Jones et al., 2007 [9]	“To obtain the smoothest surface (…) composite should be finished dry”	x	
Kaminedi et al., 2014 [13]	“Finishing and polishing under coolant resulted in the best surface smoothness and hardness values in microhybrid composite”; “Dry finishing and polishing gave the best smoothness and hardness values in nanohybrid composite”	x	x
Nasoohi et al., 2017 [14]	“Dry finishing and polishing increases the microhardness and surface roughness of microhybrid and nanohybrid composite resins”		x
de Freitas et al., 2019 [15]	“Irrigation during finishing/polishing influences the color stability and roughness of composites. The finishing/polishing protocols with abrasive discs + multilaminated + spirals and spirals with irrigation were more effective”		x

**Table 5:** Modified Consolidated Standards of Reporting Trials (CONSORT) checklist for reporting in vitro studies of dental materials.

Item	Studies					
	Dodge et al., 1991 [11]	Cardoso et al., 2006 [12]	Jones et al., 2007 [9]	Kaminedi et al., 2014 [13]	Nasoohi et al., 2017 [14]	Freitas et al., 2019 [15]
1 Abstract	Yes	Yes	Yes	Yes	Yes	Yes
2a Introduction (Background)	Yes	No	Yes	Yes	Yes	Yes
2b Introduction (Objectives)	No	Yes	Yes	Yes	Yes	Yes
3 Methods (Intervention)	Yes	Yes	Yes	Yes	Yes	Yes
4 Methods (Outcomes)	Yes	Yes	Yes	Yes	Yes	Yes
10 Methods (Statistical Methods)	No	Yes	No	Yes	Yes	Yes
11 Results (Outcomes and estimation)	Yes ^a^	Yes ^a^	Yes ^a^	Yes ^a^	Yes ^a^	Yes ^a^
12 Discussion (Limitations)	No	No	No	No	No	Yes
13 Other information (Funding)	No	No	No	No	No	Yes
14 Other information (Protocol)	No	No	No	No	No	No

^a^ No confidence interval presented.
